# A Criticism of Homœopathy

**Published:** 1909-05-15

**Authors:** J. H. Heaney

**Affiliations:** Box, Wilts


*To the Editor of* The Hospital.

### "A CRITICISM OF HOMŒOPATHY."

*To the Editor of* The Hospital.

Sir,—The pity of seeing such evident culture as is shown in Mr. Wheeler's letter under the above heading wasted through loyalty to a discredited generalisation, and the hope that he will reconsider his position must be my excuse for what is little more than a repetition of some facts clearly set forth in your recent leader.

"*Like Cures Like.*"—It will be granted that a generalisation involving the momentous issues of life or death, to be accepted or indeed true, must be applicable to every particular case. Whether vaccine therapy supports this homœopathic dictum we need not discuss. Vaccine therapy is rational. It deals with the recognised and proven *cause* of disease—with the removal or disablement of that cause. It has to do with the infinite law of cause and effect. That is the common-sense view and the truth of the matter. Why instance it in support of a generalisation ("Like cures like") that will not hold water in the following apposite case:—

The presence of lead in certain quantity in the body gives rise to a disease with definite symptoms—paralysis of certain muscles and constipation, with severe colicky pains. If "like cures like," then lead is the remedy. And judging from similar homœopathic happenings I can well imagine that there are people who would give it, indeed, have given it.

It is difficult to think that anyone could bind himself to a principle involving such dangerous trafficking with the human body, which implies the addition of fuel to the flame, and more lead where the quantity already present is causing grave and serious mischief. This condition is, of course, properly and successfully treated by preventing any further intake of lead and promoting the removal of that present.

I am, sir, sincerely yours,

Box, Wilts, May 6, 1909. J. H. Heaney, M.B.

